# Interventions for Hiccups in Adults: A Scoping Review of Western and Eastern Approaches

**DOI:** 10.1089/pmr.2024.0109

**Published:** 2025-04-17

**Authors:** Yohei Kishi, Moe Nakawaga, Anri Inumaru, Michiko Nambu, Miwa Sakaguchi, Mayumi Murabata, Mari Matsuoka, Jun Kako

**Affiliations:** Graduate School of Medicine, Mie University, Tsu, Japan.

**Keywords:** hiccup, hiccup measures, nonpharmacological interventions, pharmacological interventions, scoping review

## Abstract

Hiccups are caused by involuntary spasms of the diaphragm and external intercostal muscles. When persistent, they can significantly reduce the quality of life. However, comprehensive reviews of available treatments and their corresponding evaluation metrics remain scarce. This scoping review aimed to comprehensively map the interventions used to treat hiccups in adults and clarify the current state of outcome measures employed in existing research. We conducted a scoping review following Preferred Reporting Items for Systematic Reviews and Meta-Analyses (PRISMA) -ScR guidelines and the framework of Arksey and O’Malley. Using PubMed, Cumulative Index to Nursing and Allied Health Literature (CINAHL), and Ichushi-web databases, we identified studies published up to June 3, 2024. The search terms included “HICCUP,” “HICCOUGH,” and “SINGULTUS.” A total of 3248 articles were identified, with 499 duplicates removed. After screening 2749 titles and abstracts, 2708 articles were excluded. Full-text reviews of 41 articles led to the exclusion of 18, resulting in 23 that met the inclusion criteria. Of these, 17 studies focused on pharmacological interventions, including baclofen, metoclopramide, methylprednisolone, and Shitei-to, while 6 studies examined nonpharmacological interventions, such as acupuncture, infrared therapy, rebreathing techniques, and cervical epidural block. Outcome measures were categorized into objective and subjective evaluations. Objective measures included complete cessation, partial cessation, frequency reduction, and time to complete cessation. Subjective measures assessed the distress caused by hiccups using patient-reported scales, such as the numerical rating scale. This scoping review identified 23 studies on hiccup interventions, including five randomized controlled trials on pharmacological agents and one study on a nonpharmacological approach. Studies included both Western and Eastern medicine, offering new perspectives on hiccup management. The outcome measures were primarily objective, with some patient-reported assessments. These findings provide a foundation for future research on hiccup treatment and evaluation methods.

## Introduction

Hiccups are characterized by sudden, involuntary, and spasmodic contractions of the diaphragm and external intercostal muscles, followed by abrupt closure of the glottis, which interrupts inhalation.^1^ Although typically transient and harmless, ∼4000 hospitalized cases of hiccups are reported annually in the United States,^2^ and when hiccups become persistent, they can severely impact the patient’s quality of life. Persistent hiccups may lead to insomnia, fatigue, and social inconvenience and, in rare instances, have been associated with mortality.^3^ Particularly in patients with serious illnesses such as cancer, hiccups become a critically important issue from a palliative care perspective. Consequently, appropriate management strategies are required.

Management of hiccups broadly encompasses pharmacological and nonpharmacological interventions. Pharmacological interventions are commonly employed in clinical practice. For example, chlorpromazine, a first-generation antipsychotic, is considered to alleviate hiccups by blocking central dopaminergic receptors.^4^ Baclofen, a Gamma-Aminobutyric Acid Type B receptor agonist approved for controlling spasticity, is believed to exert antihiccup effects by inhibiting dopamine release.^5^ Metoclopramide, which has dopamine antagonist and serotonergic properties, has been reported to be among the most effective agents for terminating hiccups.^6^ Although other medications have also been suggested to be effective, the overall evidence remains limited. Nonpharmacological interventions such as vinegar ingestion, acupuncture, and certain nerve-based procedures have been reported primarily as case studies and thus lack robust evidence.^7–8^

Current evaluation methods for hiccup improvement include objective measures, such as the presence or absence of hiccup cessation,^9^ and subjective measures that capture patient distress.^8^ However, no standardized evaluation methods have been established.

This study aimed to conduct a comprehensive literature search for both pharmacological and nonpharmacological interventions for hiccups. We sought to summarize current management strategies and clarify the outcome measures for evaluating hiccup interventions.

## Methods

This scoping review aimed to comprehensively examine interventions for hiccups administered to adult patients. The review followed a standard methodological framework, adhering to PRISMA-ScR guidelines^10^ and Arksey and O’Malley’s framework.^11^ The review protocol was registered before data extraction (University Hospital Medical Information Network, UMIN000054537).

### Identifying the research question

We conducted a systematic literature search to identify interventions for hiccups in adult patients. This review was guided by two primary research questions:

1. What types of interventions for the treatment of hiccups have been studied?
2. How were improvements in hiccup evaluated in existing research?

Identifying relevant studies: A literature search was conducted using PubMed, CINAHL, and Ichushi-web, and studies that met the eligibility criteria were included. The search period covered the inception of each database until June 3, 2024. The search terms “HICCUP,” “HICCOUGH,” or “SINGULTUS” were used. The search strategy was initially developed for PubMed and was subsequently adapted for the other databases. The eligibility criteria were as follows.

• 18 years or older
• interventions for hiccups
• randomized controlled trials (RCTs), non-RCTs, single-arm trials, and retrospective cohort studies.
• written in English or Japanese

The exclusion criterion was studies with fewer than 10 participants.

### Selecting studies

The study selection process comprised two stages. In the first stage, titles and abstracts were screened, and in the second stage, full-text articles were evaluated. Both stages were independently conducted by two (Y.K. and M.N.). Disagreements between the reviewers were resolved through discussion, and if a consensus could not be reached, a third party was consulted.

### Charting the data

A standardized data extraction form was developed to capture the characteristics of each study, including the first author name, publication year, country of publication, language, journal, study design, study objectives, participant details (number of participants, sex, age, and underlying conditions), intervention methods for hiccups, evaluation methods for hiccups, and outcomes. After data extraction, the entries were reviewed and verified by an independent researcher (N.M.).

### Collating, summarizing, and reporting the results

The included studies were categorized into pharmacological and nonpharmacological interventions for hiccups. Subsequently, the studies were classified based on their design and tabulated. Finally, all outcome measures used to evaluate hiccups across the studies were extracted and tabulated. Consistent with the methodology of a scoping review, no quality or risk of bias assessments were conducted in this study.

### Patient and public involvement statement

The patients and the public were not involved in the design of this study, data collection and analysis, decision to publish, or preparation of the article.

## Results

**FIG. 1.**
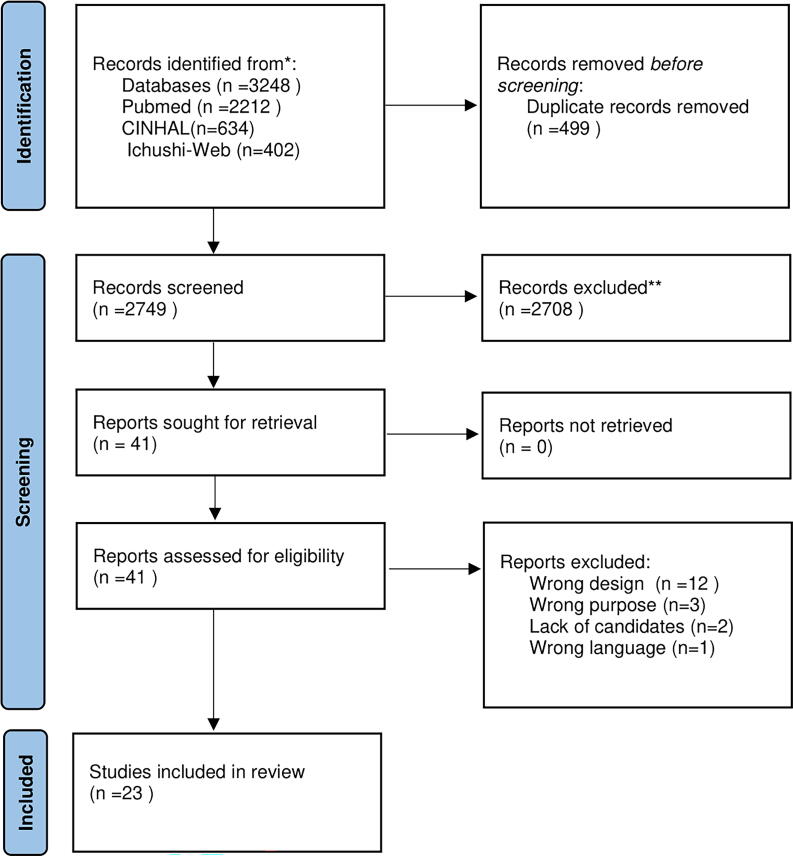
PRISMA flow diagram.

Figure 1 illustrates the screening process. A total of 3248 articles were initially identified based on the inclusion criteria. After removing 499 duplicates, the titles and abstracts of 2749 articles were screened, leading to the exclusion of 2708 articles. Full-text reviews were conducted on the remaining 41 articles and 18 were excluded due to the following reasons: inappropriate study design (*n* = 12), ineligible study objectives (*n* = 3), insufficient sample size (*n* = 2), and language incompatibility (*n* = 1). Consequently, 23 articles met the inclusion criteria and were included in this review.

Table 1 presents the characteristics of the included studies. When examining the publication timeline, the number of studies showed an increasing trend from the 1990s to the present. Geographically, studies were conducted in Japan,^12–18^ China,^19–24^ Korea,^25–27^ the United States,^28–29^ and other countries.^30–34^ The total sample size across the 23 studies was 1820 individuals, with ∼70% (1268 individuals) being male. Among the underlying conditions documented, cancer was the most frequently reported, appearing in 13 of the included studies^12–16,18–20,26–29,31^ and affecting 919 patients.

**Table 1.** Study Characteristics (*n* = 23)

Study characteristics	*N* (％)
Year of publication	
1991–2000	2 (8.7)
2001–2010	6 (26.1)
2011–2020	11 (47.8)
2021–2024	5 (21.7)
Study design	
Randomized controlled trials	6 (26.1)
Nonrandomized controlled trials	1 (4.3)
Mixed-methods study	1 (4.3)
Single-group intervention studies	6 (26.1)
Retrospective cohort studies	9 (39.1)
Country of publication	
Japan	7 (30.4)
China	6 (26.1)
Korea	3 (13.0)
United States	2 (8.7)
Germany	1 (4.3)
Italy	1 (4.3)
Iran	1 (4.3)
Israel	1 (4.3)
Taiwan	1 (4.3)

Table 2 categorizes the included studies into pharmacological and nonpharmacological interventions for hiccups, further organizing them according to study design. Seventeen studies focused on pharmacological interventions,^12–17,19–21,26–33^ among which five were RCTs. The RCTs investigated the use of baclofen,^21^ metoclopramide,^20,33^ methylprednisolone,^26^ and a combination of ephedrine and lidocaine.^30^ Although not RCTs, six studies on Kampo medicines, such as Shitei-to and Shakuyaku-Kanzoto, were reported from Asia.^12–17^ Similarly, six studies examined nonpharmacological interventions^18,22–25,34^ from Asia. Among these, only one was an RCT that evaluated a combination of acupuncture and cupping therapy.^23^

**Table 2.** Summary of Study Design and Interventions

Study design	Treatment method	*n* (%)
Pharmacological intervention		17 (73.9)
Randomized controlled trials	Metoclopramide(2), methylprednisolone (1), baclofen (1), ephedrine and lidocaine (1)	5 (21.7)
Mixed-methods study	Baclofen (1)	1 (4.3)
Single-arm trial	Baclofen (1), Shitei (1), COB (1) ※	3 (13.0)
Retrospective cohort studies	Shitei (4), olanzapine (1), gabapentin (1), methylprednisolone (1), Shakuyaku-Kanzoto (1)	8 (34.8)
Nonpharmacological intervention		6 (26.1)
Randomized controlled trials	Acupuncture and cupping (1)	1 (4.3)
Nonrandomized controlled trials	Near-infrared irradiation (1)	1 (4.3)
Single-arm trial	Hypercapnia: rebreathing with a plastic bag (1), acupuncture (1), auricular acupuncture (1)	3 (13.0)
Retrospective cohort studies	Continuous cervical epidural block (1)	1 (4.3)

※COB, combination therapy with cisapride, omeprazole, and baclofen.

Table 3 summarizes the outcome measures used to evaluate improvements in hiccup symptoms, categorized into objective and subjective assessments. Objective measures included complete cessation of symptoms, which was the most commonly used indicator^12–34^ followed by partial cessation.^12,14–16,20,21,23,24,28,31,32,34^ Frequency reduction, assessed as a continuous variable, was also reported.^19,24^ Additionally, some studies incorporated measures such as duration of hiccups^13,19,27,30^ and the presence or absence of recurrence.^18–20,23,25^ Subjective measures used to evaluate the distress caused by hiccups included the numerical rating scale (NRS), state anxiety scale (SAS), and hiccup assessment index (HAI).^24,26,27,31,32^

**Table 3.** Outcome Measures for Hiccup Improvement

Author/year	Underlying conditions	Objective outcome				Subjective outcome	Other outcome
		Complete cessation	Partial cessation	Frequency reduction	Time to complete cessation	NRS/HAI/SAS	Others
Pharmacological intervention							
Ehret CJ/2024	35% (106 patients): by the medication	○	○				
Mei M/2023	Chemotherapy for cancer	○		Remission: “More than 50% reduction in frequency compared with baseline”	Cure: Complete cessation within three days		Recurrence
Ehret CJ/2022	Cisplatin-based chemotherapy for cancer	○					Prescription of other agents
Kamoshida S/2021	Lung cancer undergoing chemotherapy	○	○				
Ōno, R/2020	Man with chemotherapy for cancer	○			Marked Effect: Complete cessation within two days Effective: Complete cessation taking more than three days		
Hosoya R/2019	44% (65): Cancer 13％ (20): Brain hemorrhage	○	○				
Yamaoka H/2018	33% (9): Neurological disorders 22％ (6): Chemotherapy for cancer	○	○				
Bahadoori A/2018	Undergoing gynecological surgery	○			Time to cessation of hiccups measured		
Go SI/2017	Chemotherapy for Cancer	○				NRS	
Wang T/2014	47％ (17): Cancer 39％ (14): Stroke 14 (5): Brain tumor	○	○				Cure： No recurrence for one week
Zhang C/2014	Stroke	○	○				
Lee GW/2013	Cancer patients with dexamethasone-induced hiccups	○			Time to hiccup cessation measured	NRS	
Porzio G/2010	Advanced cancer	○	○			NRS	
Hosomi, K/2005	68% (73): Cancer	○	○				
Saito M/2001	Caused by structural abnormalities	○			Marked effect: Complete cessation within two days Effective: Complete cessation taking more than three days Measurement: Time to cessation of hiccups		
Petroianu G/1997	previous treatments were ineffective	○	○			SAS	
Stav A/1992	Short gynecological procedures	○					
Nonpharmacological intervention							
Obuchi T/2020	Not limited to a specific disease	○					Recurrence
Xu J/2019	After joint replacement surgery	○				HAI	
Kim JE/2018	68% (19): Gastrointestinal disorders	○					Cure： No recurrence for over 48 hours
Chang CC/2008	Central nervous system disorders or those with peptic ulcers	○	○				
Hongliang X/2006	Cerebrovascular disorders	○	○				
Kou S/2005	29% (11): Inappropriate diet 26％ (10): Surgery 18％ (7): Blood transfusion	○	○	Improved: More than 50% decrease in frequency			

HAI, hiccup assessment index; NRS, numerical rating scale; SAS, state anxiety scale.

## Discussion

This review comprehensively examined pharmacological and nonpharmacological interventions for hiccups and identified three key findings. First, studies on both pharmacological and nonpharmacological interventions included a limited number of RCTs. Second, while most studies focused on Western medicine, some also reported interventions from traditional Eastern medicine. Third, by categorizing outcome measures into objective and subjective assessments, it became clear that objective measures were predominantly used, although some studies also incorporated subjective measures.

This review revealed a lack of RCTs on both pharmacological and nonpharmacological interventions. Seventeen studies on pharmacological interventions were identified,^12–17,19–21,26–33^ among which five RCTs were included. These RCTs evaluated baclofen,^21^ metoclopramide,^20,33^ and methylprednisolone^26^ and compared ephedrine and lidocaine.^30^ Notably, metoclopramide was examined in two RCTs,^20,33^ and baclofen was evaluated not only in an RCT but also in several single-arm studies.^19,21,28^ These drugs are expected to improve hiccup symptoms through mechanisms involving both the central and peripheral nervous systems, such as suppressing dopamine release, regulating vagal nerve activity, inhibiting involuntary diaphragmatic contractions, and preventing the relaxation of the lower esophageal sphincter. However, despite their potential effectiveness, side effects such as sedation, dizziness, and gastrointestinal discomfort remain important considerations because these adverse reactions have been reported with some agents.^5,6,27,35–39^ Moreover, the delayed onset of action of these medications means that hiccups persist until the effects take hold, posing a challenge in clinical practice. In fact, many study participants were receiving treatment for serious illnesses in clinical settings, with over half of the participants in pharmacological intervention studies being patients with cancer. According to a previous study, patients with cancer are more prone to developing hiccups, suggesting that hiccup management may be an important aspect of cancer treatment.^40^ These aspects should be carefully considered when considering pharmacological interventions for hiccups.

This review identified six studies,^18,22–25,34^ of which only one was an RCT. The RCT evaluated combined intervention using acupuncture and cupping therapy.^23^ Notably, acupuncture has been extensively studied in China for symptom relief, and three prospective studies on acupuncture were identified in this review. According to the Consolidated Framework for Implementation Research,^41^ the feasibility of treatments should be evaluated based on multiple factors, including invasiveness, complexity, compatibility, available resources, access to knowledge and information, and expectation of effectiveness. For instance, while acupuncture is considered potentially effective, it is highly invasive, procedurally complex, and requires access to trained practitioners, which may limit its broader application in clinical practice. Regarding invasiveness and access to medical resources, a study that used the forced inspiratory suction and swallowing tool (FISST) was reported,^42^ although it was excluded from this review because of its cross-sectional study design. FISST is a simple and minimally invasive method that involves using a specially designed straw device to drink water. Such interventions, characterized by high clinical applicability, warrant further research to explore their potential for managing hiccups effectively.

As a second key finding, this review identified several treatments derived from Eastern medicine, a topic that has not been sufficiently addressed in previous reviews.^7,43^ In contrast, most pharmacological therapies are based on Western medicine and are widely used as mainstream treatments in clinical practice. Geographically, seven studies were from Japan,^12–18^ six from China,^19–24^ three from Korea,^25–27^ and one from Taiwan.^34^ These studies included Kampo medicines such as Shitei-to and Shakuyaku-Kanzoto,^12–17^ as well as nonpharmacological interventions such as acupuncture, near-infrared irradiation, rebreathing techniques utilizing hypercapnia, and cervical epidural blocks.^18,22–25,34^ These findings suggest that hiccup research may be influenced by the widespread use of traditional medicine, complementary and alternative therapies, region-specific clinical practices, and the availability of medical resources in Asia. Eastern medical approaches offer mechanisms of action that differ from those of Western medicine, providing potential complementary treatment options. For instance, Kampo medicines emphasize restoring overall bodily balance, while acupuncture aims to alleviate symptoms through neural reflexes. In contrast, Western medical treatments often focus on directly controlling specific neural transmissions or muscle activities. Combining these distinct perspectives may offer more effective strategies for hiccup management compared with the individual treatments.

We extracted all hiccup-related outcome measures used in the included studies and categorized them into objective and subjective assessments. The results showed that all studies employed at least one objective measure, such as complete cessation,^12–34^ partial cessation,^12,14–16,20,21,23,24,28,31,32,34^ or other related endpoints. Objective measures were reported as the primary assessment tools in previous reviews on hiccups.^9^ Meanwhile, some studies introduced subjective scales, such as the HAI,^22^ NRS,^26,27,31^ and SAS,^32^ which primarily use an 11-point scale (0 = no distress; 10 = worst distress) to capture patients’ self-reported experiences. Hiccups are inherently subjective symptoms, and their severity and distress may vary among individuals. For such subjective symptoms, the use of patient-reported outcomes has been emphasized as important in previous research and international guidelines.^44,45^ Therefore, integrating both objective and subjective measures in future hiccup research has the potential to broaden the understanding of its clinical context and provide a more comprehensive reflection of patients’ experiences.

This study had several strengths. First, it comprehensively addressed both pharmacological and nonpharmacological interventions, providing a holistic perspective on hiccup treatment. Second, the extracted evaluation measures were systematically organized into tables to clearly visualize the current state of hiccup evaluation methods. This revealed the diverse range of measures being used and offered valuable insights to assist researchers in selecting appropriate indicators for measuring treatment effectiveness and working toward standardizing evaluation criteria. However, this study had several limitations. First, it included only studies published in English and Japanese, excluding relevant literature in other languages. Additionally, studies with fewer than 10 participants were excluded, which may have led to the omission of diverse approaches influenced by regional or cultural contexts. Second, as this was a scoping review, it did not qualitatively assess the included studies. Consequently, the quality of evidence and potential biases in the literature were not evaluated. Third, due to significant variability in the diseases studied, this review did not conduct analyses by disease type. Therefore, this review may not fully encompass all existing approaches to hiccup management, which should be considered when interpreting the results.

## Conclusion

This scoping review mapped both pharmacological and nonpharmacological interventions for hiccups and identified 23 studies. Among these, five RCTs examined pharmacological agents, such as metoclopramide, baclofen, and methylprednisolone, and compared ephedrine and lidocaine. One RCT evaluated a nonpharmacological approach in which acupuncture and cupping therapy were combined. Studies were reported from Western and Eastern medicine, providing new perspectives on hiccup management. By extracting and categorizing outcome measures into objective and subjective assessments, this review revealed that objective endpoints were predominantly utilized, although some studies also employed patient-reported measures. The findings presented here summarize existing research on hiccup interventions and their evaluation methods, providing a foundational resource for future research.

## Data Availability

All data relevant to the study are included in the article.

## Abbreviations Used

CINAHL: Cumulative Index to Nursing and Allied Health Literature
FISST: Forced Inspiratory Suction and Swallow Tool
GABAB: Gamma-Aminobutyric Acid Type B
HAI: Hiccup Assessment Instrument
NRS: Numerical Rating Scale
PRISMA: Preferred Reporting Items for Systematic Reviews and Meta-Analyses
RCT: Randomized Controlled Trial
SAS: Subjective Assessment Scale
UMIN: University Hospital Medical Information Network
